# Supplementary material for: Long-term genomic coevolution of host-parasite interaction in the natural environment
Source: Nat Commun. 2017 Jul 24;8:111. doi: 10.1038/s41467-017-00158-7 (PMC5524643; doi:10.1038/s41467-017-00158-7)
Supplement: Supplementary file 1 — Supplementary Information [file 41467_2017_158_MOESM1_ESM.pdf]

File name: Supplementary Information
Description: Supplementary Figures, Supplementary Tables, Supplementary Discussion and Supplementary References

File name: Supplementary Data 1
Description: CRISPR spacer sequences and Blast hits.

File name: Peer Review File
Description:

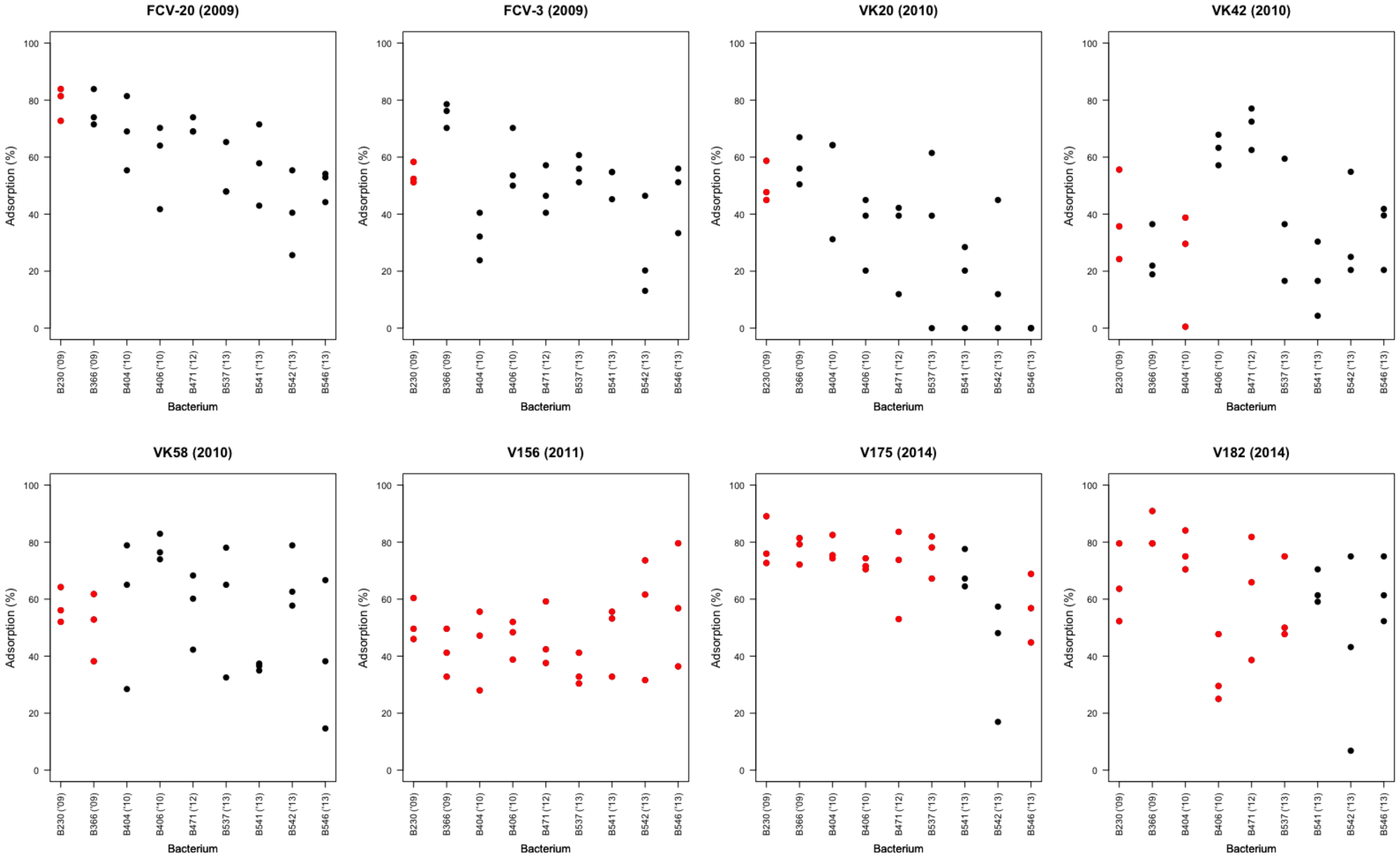

Supplementary Figure 1. Percentage of phage adsorption on bacterial hosts isolated in different years. Red dots represent (individual values of) adsorption on susceptible hosts (see Fig. 2 in the main text) and black dots adsorption on resistant hosts. Assay was done in three replicates.

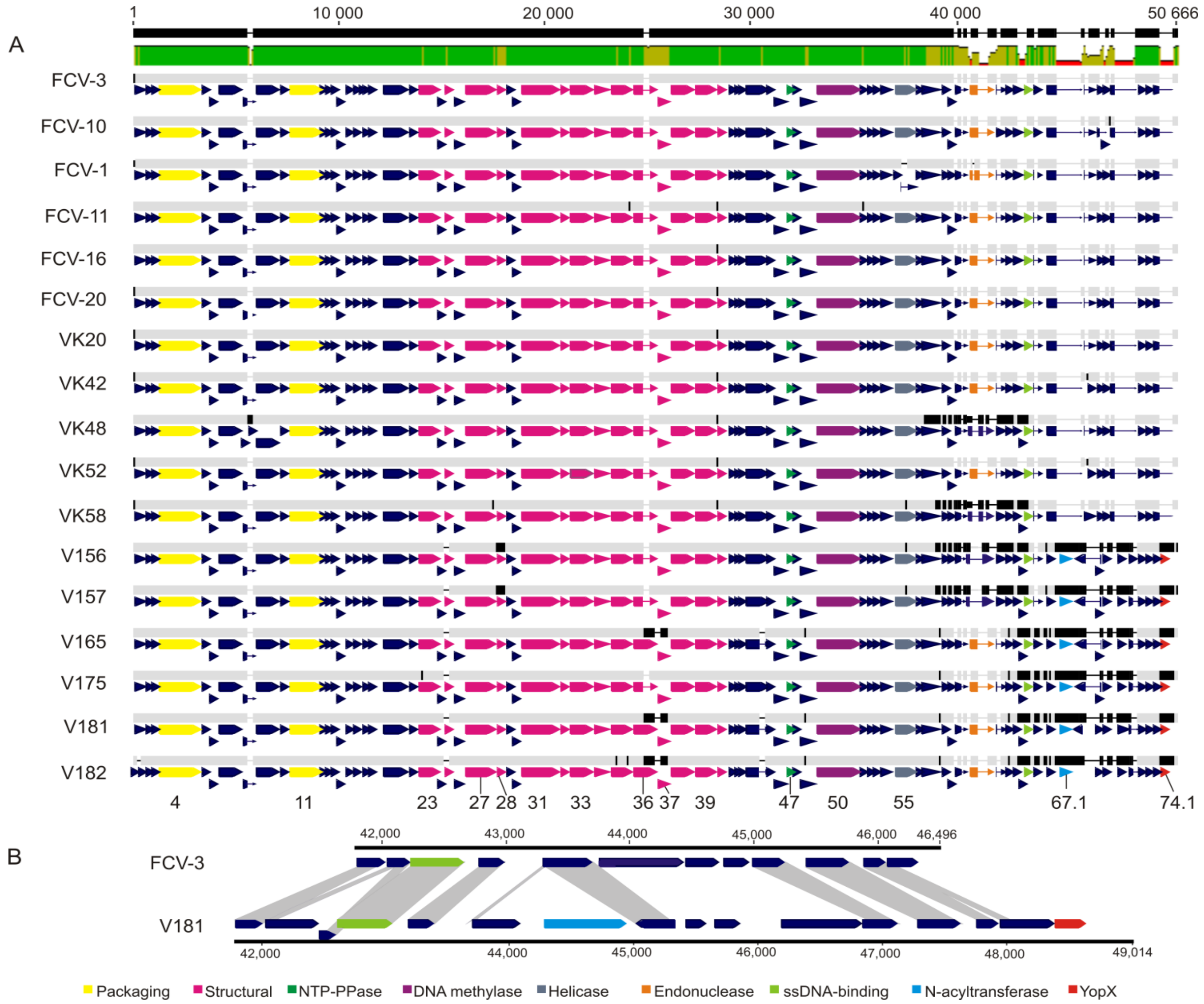

Supplementary Figure 2. (**A**) The genome alignment shows predicted open reading frames (ORFs). Numbers underneath the alignment indicate the assigned number of some of the ORFs. ORFs with putative function are marked with colors indicating the functions shown in the bottom of **B**. In the consensus sequence bar above the genomes, green color indicates 100% identical DNA-sequences, yellow 30% and red < 30% identity respectively. Note that also the bar height changes accordingly. (**B**) A closeup to the end of genomes FCV-3 and V181 where the 100% identity between the ORFs is shown in grey.

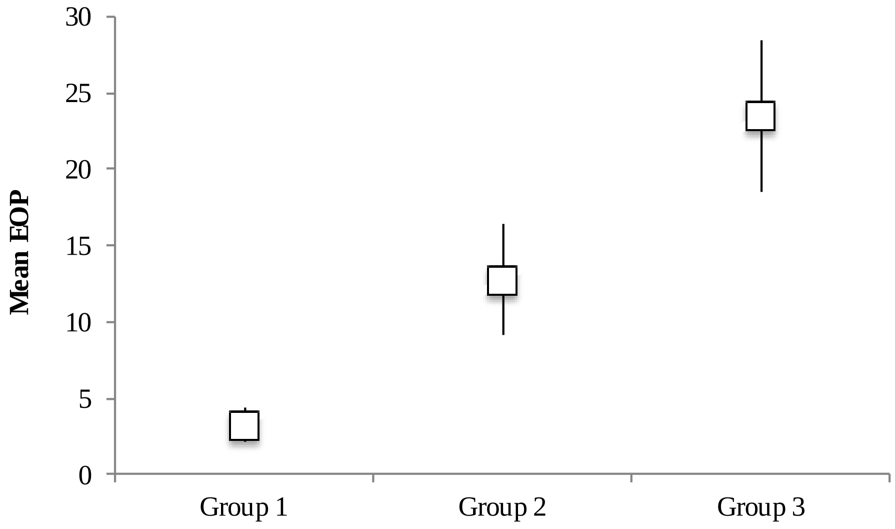

Supplementary Figure 3. Mean efficiency of plating (EOP, phage titers in relation to host B230, Supplementary Table 3) of phages belonging to genomic group 1, 2 and 3 (see Fig. 2 in the main text) in all bacterial hosts (+/- S.E.M.)

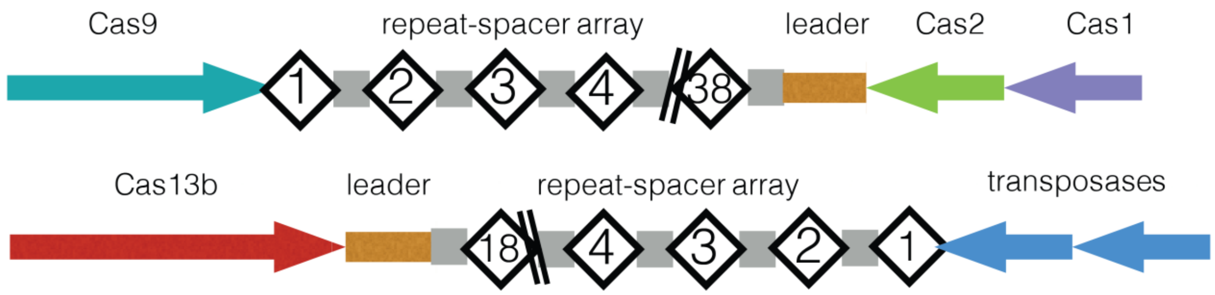

Supplementary Figure 4. Diagrams of CRISPR1 (top) and CRISPR2 (below) loci.

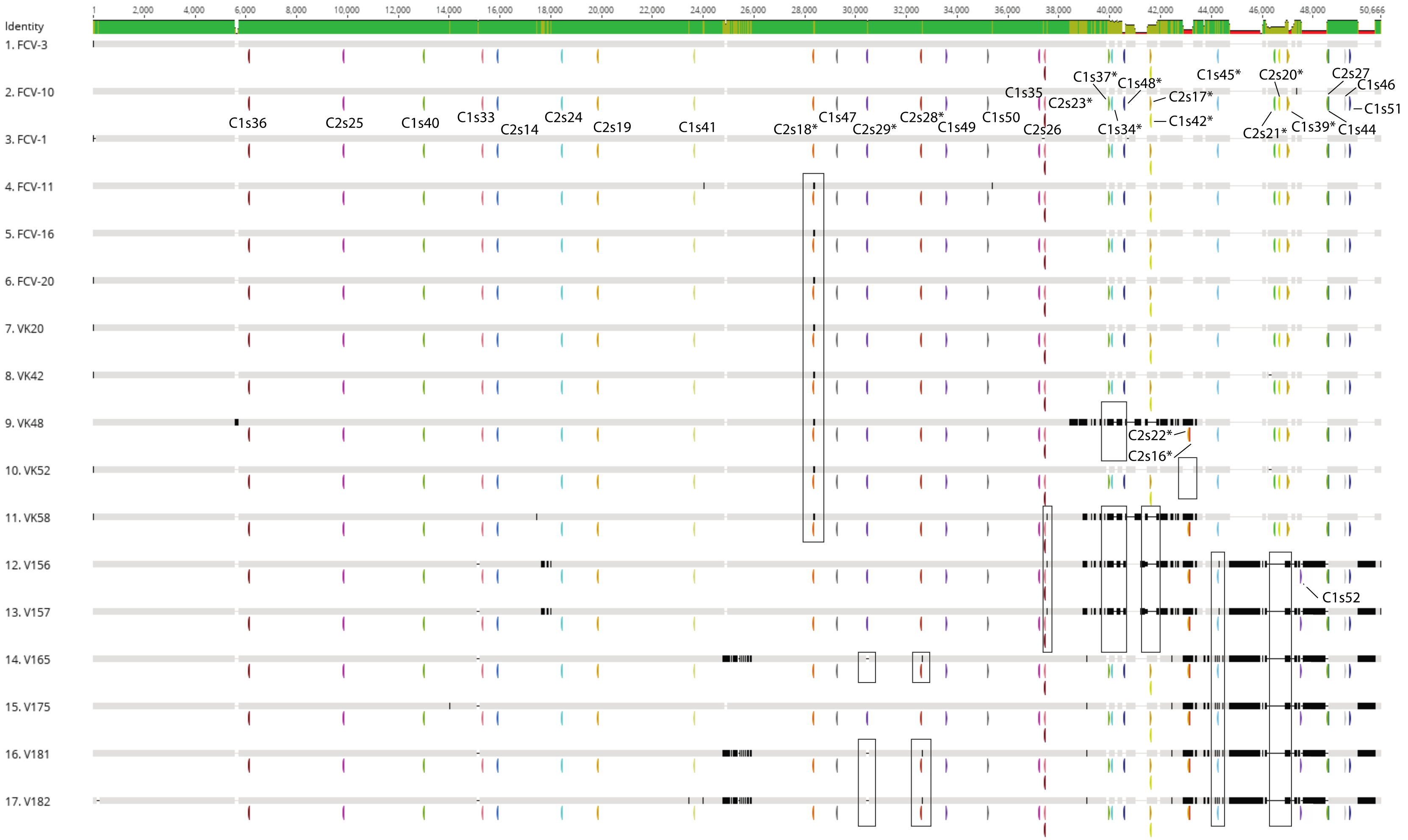

Supplementary Figure 5. Location of protospacers in the phage genomes. Protospacers that have variation among the phage genomes are marked with an asterisk and these corresponding changes are marked with rectangles in the protospacer areas (one rectangle may include multiple protospacers). Color coding only highlights similar protospacers in different phages and does not correspond to colors in Fig. 2a.

Supplementary Table 1. *Flavobacterium columnare* isolates used in this study. All isolates belong to the genetic group C.

Bacterial isolate	Date of isolation	Source
B425	14th August 2007	rainbow trout
B447	14th August 2007	tank water
B230	30th June 2009	outlet water
B235	7th July 2009	tank water
B236	7th July 2009	tank water
B237	7th July 2009	tank water
B245	14th July 2009	tank water
B339	5th July 2010	outlet water
B366	2nd August 2010	outlet water
B397	21st June 2010	inlet water
B404	2nd August 2010	inlet water
B406	16th August 2010	inlet water
B471	12th July 2012	rainbow trout
B537	28th June 2013	rainbow trout
B541	8th August 2013	rainbow trout
B542	8th August 2013	rainbow trout
B546	8th August 2013	rainbow trout
C1	1997	Reference 1

Supplementary Table 2. Bacteriophage isolates used in this study

Phage isolate	Date of isolation	Source	Enrichment host	Enrichment host isolation year	Phage genome size (bp)	Accession Number
FCV-2	7th July 2009	tank water	B235	2009		
FCV-3	7th July 2009	tank water	B236	2009	46 496	KY951963
V99	7th July 2009	tank water	B237	2009		
FCV-9	14th July 2009	tank water	B245	2009		
FCV-10	14th July 2009	tank water	B247	2009	46 482	KY979236
FCV-1	22nd July 2009	tank water	C1	1997	46 450	KY979235
FCV-11	22nd July 2009	outlet water	C1		46 481	KY951964
FCV-16	25th August 2009	outlet water	C1		46 481	KY979237
FCV-20	11th August 2009	outlet water	C1		46 469	KY979238
VK16	5th July 2010	inlet water	C1			
VK20	5th July 2010	tank water	C1		46 496	KY979243
VK42	2nd August 2010	tank water	B339	2010	46 455	KY979244
VK46	2nd August 2010	tank water	C1			
VK48	2nd August 2010	tank water	C1		46 570	KY979245
VK51	16th August 2010	inlet water	C1			
VK52	16th August 2010	tank water	C1		46 455	KY979246
VK53	30th August 2010	outlet water	C1			
VK54	30th August 2010	eathren pond	C1			
VK58	11th October 2010	outlet water	C1		46 448	KY979247
V156	4th August 2011	eathren pond	C1		48 564	KY979239
V157	4th August 2011	inlet water	B270	2009	48 565	KY979240
V158	4th August 2011	inlet water	C1			
V162	14th August 2014	outlet water	B366	2010		
V165	14th August 2014	inlet water	B366		49 013	KY979241
V167	214th August 014	outlet water/river	B366			
V171	22th August 2014	inlet water	B366			
V175	22nd August 2014	outlet water	B366		49 016	KY992519
V178	222nd August 014	outlet water/river	B366			
V181	27th August 2014	inlet water	B366		49 014	KY992520
V182	27th August 2014	outlet water	B366		49 099	KY979242

Supplementary Table 3. (**A**) Phage plaque numbers in bacterial hosts and (**B**) efficiency of plating (EOP) calculated using strain B230 as a reference host.

A)

Phage	B425	B447	B230	B235	B236	B237	B245	B339	B366	B397	B404	B406	B471	B537	B541	B542	B546
FCV-2	0	0	1E+07	1E+07	1E+07	1E+08	1E+07	0	0	0	0	0	0	0	0	0	0
FCV-3	0	0	1E+07	1E+07	1E+07	1E+08	1E+07	0	0	0	0	0	0	0	0	0	0
V99	0	0	1E+07	1E+07	1E+07	1E+08	1E+07	0	0	0	0	0	0	0	0	0	0
FCV-9	0	0	1E+08	1E+08	1E+08	1E+07	1E+08	0	0	0	0	0	0	0	0	0	0
FCV-10	0	1E+06	1E+08	1E+08	1E+08	1E+07	1E+08	1E+06	0	0	0	0	0	0	0	0	0
FCV-1	0	1E+06	1E+08	1E+08	1E+08	1E+09	1E+08	1E+06	0	0	0	0	0	0	0	0	0
FCV-11	0	1E+06	1E+06	1E+06	1E+06	1E+06	1E+06	0	0	1E+06	0	0	0	0	0	0	0
FCV-20	0	1E+09	1E+07	1E+07	1E+08	1E+08	1E+07	0	0	1E+08	0	0	0	0	0	0	0
FCV-16	0	1E+07	1E+06	1E+06	1E+06	1E+07	1E+07	0	0	1E+07	0	0	0	0	0	0	0
VK16	0	1E+07	1E+05	1E+05	1E+05	1E+06	1E+06	0	0	1E+07	0	0	0	0	0	0	0
VK20	0	1E+07	1E+05	1E+06	1E+06	1E+06	1E+06	0	0	1E+07	0	0	0	0	0	0	0
VK42	0	1E+07	1E+07	1E+06	1E+07	1E+06	1E+06	1E+08	0	1E+07	1E+08	0	0	0	0	0	0
VK46	0	1E+08	1E+07	1E+06	1E+07	1E+06	1E+06	0	0	1E+08	0	0	0	0	0	0	0
VK48	0	1E+08	1E+07	1E+07	1E+07	1E+07	1E+07	1E+07	0	1E+09	0	0	0	0	0	0	0
VK51	0	1E+07	1E+07	1E+06	1E+06	1E+07	1E+07	0	1E+07	1E+07	0	0	0	0	0	0	0
VK52	0	1E+07	1E+07	1E+07	1E+07	1E+08	1E+07	1E+07	0	1E+07	1E+08	0	0	0	0	0	0
VK53	0	1E+07	1E+07	1E+05	1E+06	10000	1E+05	0	0	1E+08	0	0	0	0	0	0	0
VK54	0	1E+06	1E+07	1E+06	1E+07	1E+07	1E+06	0	1E+07	1E+06	0	0	0	0	0	0	0
VK58	0	1E+06	1E+07	1E+06	1E+07	1E+07	1E+06	0	1E+08	1E+08	0	0	0	0	0	0	0
V156	1000	1E+08	1E+08	1E+07	1E+08	1E+09	1E+08	1E+09	1E+09	1E+09	1E+09	1E+08	1E+09	1E+09	1E+06	0	1E+07
V157	0	1E+06	1E+05	10000	1E+05	1E+06	1E+06	1E+07	1E+07	1E+07	1E+07	1E+06	1E+07	1E+07	0	0	1E+06
V158	0	1E+08	1E+08	1E+07	1E+09	1E+08	1E+08	1E+09	1E+09	1E+09	1E+09	1E+09	1E+09	1E+09	1E+06	0	1E+07
V162	1E+08	1E+08	1E+07	1E+07	1E+09	1E+09	1E+07	1E+08	1E+09	1E+08	1E+09	1E+09	1E+09	1E+09	1E+08	1E+08	0
V165	1E+08	1E+09	1E+07	1E+07	1E+09	1E+09	1E+07	1E+08	1E+09	1E+08	1E+09	1E+09	1E+09	1E+09	1E+07	1E+08	0
V167	1E+08	1E+09	1E+07	1E+07	1E+09	1E+09	1E+08	1E+08	1E+09	1E+09	1E+08	1E+09	1E+09	1E+09	1E+06	1E+08	0
V171	1E+08	1E+09	1E+06	1E+07	1E+07	1E+08	1E+06	1E+07	1E+09	1E+09	1E+08	1E+09	1E+09	1E+09	1E+07	1E+08	0
V175	0	1E+09	1E+09	1E+07	1E+09	1E+09	1E+08	1E+09	1E+09	1E+09	1E+08	1E+09	1E+09	1E+09	0	0	1E+07
V178	0	1E+09	1E+09	1E+07	1E+09	1E+09	1E+08	1E+09	1E+09	1E+09	1E+08	1E+09	1E+08	1E+09	0	0	1E+07
V181	1E+07	1E+09	1E+07	1E+06	1E+08	1E+08	1E+07	1E+09	1E+09	1E+09	1E+08	1E+09	1E+09	1E+09	0	0	0
V182	1E+08	1E+09	1E+09	1E+06	1E+09	1E+09	1E+07	1E+09	1E+09	1E+09	1E+09	1E+09	1E+09	1E+09	0	0	0

B)

Phage	B425	B447	B230	B235	B236	B237	B245	B339	B366	B397	B404	B406	B471	B537	B541	B542	B546
FCV-2	0	0	1	1	1	10	1	0	0	0	0	0	0	0	0	0	0
FCV-3	0	0	1	1	1	10	1	0	0	0	0	0	0	0	0	0	0
V99	0	0	1	1	1	10	1	0	0	0	0	0	0	0	0	0	0
FCV-9	0	0	1	1	1	0,1	1	0	0	0	0	0	0	0	0	0	0
FCV-10	0	0,01	1	1	1	0,1	1	0,01	0	0	0	0	0	0	0	0	0
FCV-1	0	0,01	1	1	1	10	1	0,01	0	0	0	0	0	0	0	0	0
FCV-11	0	1	1	1	1	1	1	0	0	1	0	0	0	0	0	0	0
FCV-20	0	100	1	1	10	10	1	0	0	10	0	0	0	0	0	0	0
FCV-16	0	10	1	1	1	10	10	0	0	10	0	0	0	0	0	0	0
VK16	0	100	1	1	1	10	10	0	0	100	0	0	0	0	0	0	0
VK20	0	100	1	10	10	10	10	0	0	100	0	0	0	0	0	0	0
VK42	0	1	1	0,1	1	0,1	0,1	10	0	1	10	0	0	0	0	0	0
VK46	0	10	1	0,1	1	0,1	0,1	0	0	10	0	0	0	0	0	0	0
VK48	0	10	1	1	1	1	1	1	0	100	0	0	0	0	0	0	0
VK51	0	1	1	0,1	0,1	1	1	0	1	1	0	0	0	0	0	0	0
VK52	0	1	1	1	1	10	1	1	0	1	10	0	0	0	0	0	0
VK53	0	1	1	0,01	0,1	0,001	0,01	0	0	10	0	0	0	0	0	0	0
VK54	0	0,1	1	0,1	1	1	0,1	0	1	0,1	0	0	0	0	0	0	0
VK58	0	0,1	1	0,1	1	1	0,1	0	10	10	0	0	0	0	0	0	0
V156	1E-05	1	1	0,1	1	10	1	10	10	10	10	1	10	10	0,01	0	0,1
V157	0	10	1	0,1	1	10	10	100	100	100	100	10	100	100	0	0	10
V158	0	1	1	0,1	10	1	1	10	10	10	10	10	10	10	0,01	0	0,1
V162	10	10	1	1	100	100	1	10	100	10	100	100	100	100	10	10	0
V165	10	100	1	1	100	100	1	10	100	10	100	100	100	100	1	10	0
V167	10	100	1	1	100	100	10	10	100	100	10	100	100	100	0,1	10	0
V171	100	1000	1	10	10	100	1	10	1000	1000	100	1000	1000	1000	10	100	0
V175	0	1	1	0,01	1	1	0,1	1	1	1	0,1	1	1	1	0	0	0,01
V178	0	1	1	0,01	1	1	0,1	1	1	1	0,1	1	0,1	1	0	0	0,01
V181	1	100	1	0,1	10	10	1	100	100	100	10	100	100	100	0	0	0
V182	0,1	1	1	0,001	1	1	0,01	1	1	1	1	1	1	1	0	0	0

Supplementary Table 4. Conserved domains detected in the predicted phage open reading frames

ORF	Name	Accession	Description	Interval	Blastp E-value
ORF4	Terminase_GpA	pfam05876	Phage terminase large subunit (GpA)	44-654	1.27e-100
ORF7	SNF2_N	pfam00176	SNF2 family N-terminal domain	22-278	1.00e-13
ORF8	ParB	smart00470	ParB-like nuclease domain	15-109	2.27e-08
ORF9	PAPS_reductase	cd01713	This domain is found in phosphoadenosine phosphosulphate (PAPS) reductase enzymes	32-222	3.06e-26
ORF10	STKc_TLK	cd13990	Catalytic domain of the Serine/Threonine kinase, Tousled-Like Kinase; STKs catalyze the transfer of the gamma-phosphoryl group from ATP to serine/threonine residues on protein substrates.	2-37	6.53e-03
ORF11	Phage_portal_2	pfam05136	Phage portal protein, lambda family	65-402	3.47e-33
ORF16	Peptidase_M15_4	pfam13539	D-alanyl-D-alanine carboxypeptidase	87-152	1.39e-12
ORF21	S49_Sppa_N_C	cd07023	Signal peptide peptidase A (SppA), a serine protease, has catalytic Ser-Lys dyad	76-284	6.83e-28
ORF23	Phage_cap_E	pfam03864	Phage major capsid protein E	22-357	3.93e-23
ORF27	COG4386	COG4386	Mu-like prophage tail sheath protein gpL [Mobilome: prophages, transposons]	26-482	3.54e-29
ORF31	tape_meas_TP901	TIGR01760	phage tail tape measure protein, TP901 family, core region	62-409	2.41e-19
ORF33	Baseplate_J	pfam04865	Baseplate J-like protein	69-325	6.39e-21
ORF34	DUF2313	pfam10076	Uncharacterized protein conserved in bacteria (DUF2313). Members comprise various hypothetical and putative bacteriophage tail proteins	40-152	3.04e-10
ORF39	COG4379	COG4379	Mu-like prophage tail protein gpP [Mobilome: prophages, transposons];	5-290	3.18e-11
ORF42	PRK12539	PRK12539	RNA polymerase sigma factor	16-47	9.89e-03
ORF44	COG5665	COG5665	CCR4-NOT transcriptional regulation complex, NOT5 subunit [Transcription]	95-222	4.23e-03
ORF46	RecT	pfam03837	RecT family	24-185	3.43e-03
ORF47	NTP-PPase_u3	cd11540	Nucleoside Triphosphate Pyrophosphohydrolase (EC 3.6.1.8) MazG-like domain found in a group of uncharacterized proteins from bacteria and archaea	8-98	4.86e-20
ORF50	N6_N4_Mtase	pfam01555	DNA methylase	463-713	2.63e-15
ORF54	HTH_36	pfam13730	Helix-turn-helix domain	17-72	1.54e-04
ORF55	PIF1	pfam05970	PIF1-like helicase	4-288	2.91e-27
ORF58	PRK03992	PRK03992	proteasome-activating nucleotidase	99-141	1.54e-03
ORF61*	NUMOD4	pfam07463	NUMOD4 motif	7-55	3.38e-11
ORF65	PRK05733	PRK05733	single-stranded DNA-binding protein	1-110	1.32e-16
ORF66	CE_PFGI_1_parB	TIGR03764	integrating conjugative element, PFGI_1 class, ParB family protein	12-51	6.26e-03
ORF67.1**	lipid_A_lpxD	TIGR01853	UDP-3-O-[3-hydroxymyristoyl] glucosamine N-acyltransferase LpxD	54-147	1.07e-16
ORF74.1**	YopX	pfam09643	YopX protein	5-83	1.02e-08

*not in phage genomes VK48, VK58, V156, V157
**only in phage genomes V156, V157, V175, V181 and V182

Supplementary Table 5. Characterization of CRISPR loci in *Flavobacterium columnare* strains

	**CRISPR1**	**CRISPR2**
Unique spacers	52	29
Spacers shared among all isolates	30/52 (~58%)	13/29 (~45%)
Unique spacers matching phage genomes	18/52 (~35%)	15/29 (~52%)
Phage protospacers (PP) with C1 PAM	16/18 (~89%)	8/15 (~53%)
Change in PP sequence after spacer appearance	6/18 (~33%)	9/15 (60%)
Resurfacing of ancestral PP sequence in phage genomes after disappearance of spacer	4/6 (~67%)	3/9 (~33%)
Unique spacers matching *F. columnare* genome	9/52 (~17%)	2/29 (~7%)
F. columnare protospacers with C1 PAM	6/9 (~67%)	1/2 (50%)
Spacer target strand (phage)	47% coding	100% coding
Cas genes	*Cas9, Cas1, Cas2* (type II-C)	*Cas13b* (type VI-B)
Repeat sequence	GTTGTGGTTTGATTAAAGA TTAGAAAACACGATATT	GTTGGGAAAGCCCTTATT TTGAAGGGTATCTACAAC

Supplementary Table 6. 20 nucleotides long DNA regions upstream (5' end) and downstream (3' end) of C1 protospacers using the guide-centric approach[2]. Proposed PAM-sequence marked in bold.

	C1 protospacer surrounding regions	
**Spacer**	**20 nt (5' end)**	**20 nt (3' end)**
C1s33	GTTTCGCTTCTAAGCATTGG	**AATGCTAAAA**ATGTTCATGT
C1s34	TATTTTACGCCGATAGAACA	**GATTTTAAAA**ATTATTTATT
C1s35	TTATCATAAGTTAATACAGA	**TGTTTTAAAA**AAAATTGATG
C1s36	TTTTCTGGTGGTAAAGATAG	**AGCTATAAAA**AGAGATAGAA
C1s37	TCTTTGTCGAACTTTTCTTT	**GTCTTTAAAA**ACAATTTCTT
C1s39	AGAAAATCTTCACCACCTAA	CTCATTGCATGTACTTTCAC
C1s40	TTTATAGATGGAATTGCAAG	ACGATTCAAAAAATTATACA
C1s41	TTTACTCATTAACTGTAATA	**TTAATTAAAA**AAGATTCTGG
C1s42	TGATTCATAATCTTTAATTG	**AAGTATAAAA**CAATCCTAAG
C1s44	GAAAAGTATTAAAAACTTGT	GGTTATAAATGGTTAACTAA
C1s45	AAATAAAGAAAGGTGTCGAA	**AGCRCTAAAA**AATCCTACCT
C1s46	GCAATGAAAGAACAAATCAA	**GAGTTTAAAA**GTTTATCAAA
C1s47	ATTTTGATTTGCCTTGGAGT	**AATTATAAAA**ATGGCAGAAA
C1s48	TGTTTTTTATTTAATTTTTT	**CATTGTAAAA**TATTAATTAA
C1s49	GCGTGTATTTAAAAGTTTCA	**CCTTTTAAAA**TACTACTTTC
C1s50	TTTCTATTCATTTTGATAGC	**AGGACTAAAA**ACAGTTTCTC
C1s51	TTCCAAAACCTGTTCTTTAT	**TATCATAAAA**AACAGCAATT
C1s52	TGTAAAGATTGTATTGATTT	**GGCATTAAAA**TTTTTTCCAT
WebLogo	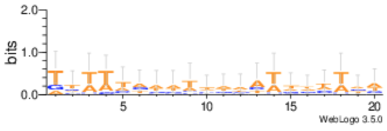	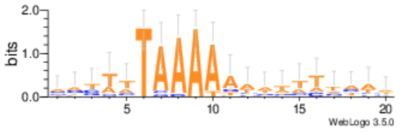

Supplementary Table 7. 20 nucleotide RNA regions (from predicted ORFs) upstream (5' end) and downstream (3' end) of C2 protospacers using the target-centric approach[2]. Sequence outside of predicted transcript in italic and sequence identical to the proposed C1 PAM in bold. Standard IUPAC codes used in cases of polymorphism between phage genomes.

	C2 protospacer surrounding regions	
**Spacer**	**20 nt (5' end)**	**20 nt (3' end)**
C2s14	CAAAGAGUUGCAGGAUGGAU	GUACUUAAAUAUAAAUAAUG
C2s16	AGUUAGAAACAAAUCCAGAG	**AGTGGUAAAA**AAAAGAGUGA
C2s17	UUAGGAUUGUUUUAUACUUU	CAAUUAAAGAUUAUGAAUCA
C2s18	GGGUGAAGUUCCUAAACCGM	**AUAAUUAAAA***UGAUAACAUU*
C2s19	GCUCAAGAACAAGCCAAAGU	AAAGUAUAAAAAAUGCUUUU
C2s20	UACAACUAUUACCUUUGAUU	**GUAUAUAAAA**UACAGAAAGC
C2s21	AAAGUAUUUUUUUAGACCCU	AAAACCUUUCAUUGAAUAUA
C2s22	UUAAGAGCCUGUUGUUUAGU	AGUUAUCAAAAAAAUAUCAA
C2s23	AAAAAUUACAAUGGCUAUAA	**AAAUAUAAAA**AACAUUGAAU
C2s24	UUAAAACUAGAGUAAAGCCA	**UGAUUUAAAA**AAUGAUGCUG
C2s25	UUGGCGGGUUUUUAUUGAUU	ACUAUUUUUUAACCCAAAGG
C2s26	AAAUCUAUUCUUUUAAAAAA	UAUUUACAAAAAAYUAAAUA
C2s27	ACCGAAAUUUUGAAAUCAAA	**AGUAUUAAAA**ACUUGUUUAG
C2s28	GAACAUUAGGAAGAAGUAUU	UUGGGGUAAAAUAAUGGAGU
C2s29	AAAAAGAGCGUGCCGAACGU	**CAAUUUAAAA**UUAGAAUAAA
WebLogo	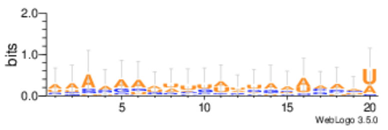	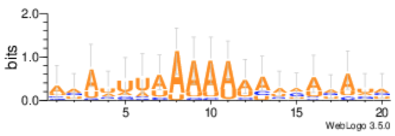

Supplementary Table 8. Spacers targeting the 11 kbp area (27 352 – 38 174 in the consensus sequence) and corresponding changes ("mutations") in the protospacers.

Spacer	Protospacer location (consensus)	Presence of spacer in bacteria	Protospacer mutations	Mutated protospacer in phages
C2s18	28 361 – 28 390	2007 (B447) and 2010 (B397)	1 SNP	2009-2010
C1s47	29 294 – 29 322		*none*	
C2s29	30 457 – 30 486	2014 (B546)	12 bp deletion	2014
C2s28	32 604 – 32 632	2014 (B546)	2 SNPs	2014
C1s49	33 591 – 33 620		*none*	
C1s50	35 230 – 35 259		*none*	
C1s35	37 247 – 37 276		*none*	
C2s26	37 472 – 37 501		*none*	
C2s23	37 487 – 37 516	2010 (B366)	1 SNP	2010 - 2011

Supplementary Table 9. PCR and sequencing primers for phage genome sequencing and amplification of *F. columnare* CRISPR loci.

	Primer	Sequence
**Phage genome**	V113Rev7	TTCCACCATTCCGAACCACC
	EndF	AATCAAAATAAAGCGATATG
	EndR	TGAATCCTTCTGCAACTTCTTTC
**CRISPR**	CRISPR1 1F	GGACGAGGTTCAACGAAGT (PCR product FWD)
	CRISPR1 2F	GATATTTAAATCATCAGTAGT
	CRISPR1 1R	CCCTAAAGCACCACAACCCA (PCR product REV)
	CRISPR1 2R	GATTTAACTATTTGTAATAT
	CRISPR1 3R	ACAACGGTAACTTTTTAAAT
	CRISPR2 1F	GGTCTAAATACAATTGCTCTTTGACATT (PCR product FWD)
	CRISPR2 1R	TTAACGCTCGTCCCTCCTTCAA (PCR product REV)

Supplementary Discussion

Phage genomes. Genome sequencing of phage isolates from 2009 to 2014 resulted in highly similar sequences with the GC-content varying from 29.7 to 30.0%. Phage genomes showed significant sequence identity to a previously described *F. columnare* phage FCL-2, suggesting that these phages have only recently diverged. Structural proteins were similar to those of marine *Cellulophaga* phages, especially phiSM, as was also reported for FCL-2[3].

For most of the predicted open reading frames (ORFs) (in phage FCV-3 52 out of 74) no putative function was assigned (Supplementary Table 3). Phage isolates from the year 2009, and three out of five of the 2010 isolates showed almost 100% sequence identity. These phages were assigned to Group 1, as shown in Fig. 2. In the two other 2010 isolates, VK48 and VK58 (Group 2), only very few differences in the nucleotide sequences in seven ORFs (56-62) could be detected, leading to only a few actual amino acid changes in ORFs 56-58 and 61-62. No putative function was predicted for these ORFs, although ORF 56 showed structural similarity to a primase-helicase of T7 and therefore could be involved in the phage replication. ORF 60 was notably different in these phages with only 14% amino acid-similarity. A homology search of the alignment of ORF 60 in FCV-3 and VK48 suggested putative function as a DNA-binding protein. The same differences were seen in the seven ORFs of the isolates from 2011, but in addition (similar to the most recent isolates from 2014 assigned to Group 3), these isolates had extra DNA in the terminal regions of the genomes, leading to increased genome size (46 448 bp in 2009 to 49 121 bp in 2014). The source of the additional DNA is not known but one possibility could be prophages, albeit none were detected during the experiments. Interestingly, the above-mentioned seven ORFs (56-62) in the 2014 isolates were identical to the 2009 isolates (except for a single nucleotide difference in ORF 61).

Single nucleotide differences resulting to change in one amino acid (glutamic acid to lysine) were seen in the 2011 isolates V156 and V157 in ORF 27. This ORF codes for a putative tail protein, having also structural similarity to a tail sheath protein. Two amino acid sequence differences were detected in the subsequent ORF 28, a putative structural protein (valine to isoleucine in both). Changes in both ORFs were identical in these two phages but were missing again in the 2014 isolates. In the 2014 phages (Group 3, except V175), changes identical between the genomes

concentrate on ORF 36 (several differences, including four additional amino acids and one missing) and ORF 37 (three amino acid changes alanine to valine, lysine to arginine and asparagine to histidine) which follow ORFs that contain predicted putative base plate domain (Blastp E-value 6.39e-21) and a tail protein domain (Blastp E-value 3.04e-10) (see Supplementary Table 4) and one hypothetical protein. As tail genes are in many phages clustered and the genes encoding base plate are in many cases by several other tail genes, it is possible that these predicted ORFs are structural. This could partly explain the expansion in host range, as mutations in tail fiber genes are typically associated changes in phage host range.

CRISPR PAM and PFS sequences. We searched for protospacer adjacent motifs (PAMS) by analysing 20 bp long regions surrounding each of the 18 C1 protospacers using the guide-centric approach[2]. Alignment of the results with WebLogo[4] revealed a putative 3' PAM with the sequence NNNNNTAAAA (Supplementary Table 6) shared by 15 of the 18 C1 protospacers. This is the longest PAM sequence described to date (10 bp) if the 5 bp linker sequence is included[2]. *Flavobacterium columnare's* C1 PAM was briefly addressed in a previous study[5], in which the authors report a similar yet truncated version of the sequence described here.

Analysing protospacer flanking sites (PFSs) associated with the 15 C2 RNA-protospacers revealed a preference for U or A in all but one 5' PFSs (Supplementary Table 7). Also, in the 5' PFS of spacer C2s18, A is replaced by C in some of the 2009-2010 phage genomes. These findings are in line with the study by Smargon *et al.*[6], showing that in type VI-B systems a 5' PFS of A and U enhance interference, while C acts as an inhibitor. In the case of 3' PFSs, either or both previously reported patterns (NAN or NNA) were found in 10 of the 15 C2 protospacers. These phage genomes are, however, high in AT-content (GC%=29.8), which contributes to making such patterns likely.

As C2 contains no known spacer-acquisition related genes, it is possible that spacers are acquired autonomously (possibly by the two transposase-like genes downstream of the repeat-spacer array) or *in trans* by utilizing Cas1 and Cas2 from the C1 locus, as suggested by Smargon *et al.*[6]. If C2 spacer acquisition was dependent on C1 activity, we would expect to detect protospacer-adjacent sequences similar to those found in C1 PAMs, since bias towards specific PAMs is also governed by the acquisition machinery[7, 8]. Surprisingly, almost half (7/15) of the C2 protospacer-adjacent sequences were identical to the proposed C1 PAM consensus NNNNNTAAAA, while the rest had more variability (see Supplementary Table 7). A possible explanation for this variability could be the lack

of selection imposed on these protospacers-adjacent sequences after acquisition. Type VI CRISPR systems are considered to be independent on PAM sequences (only relying on PFSs) [9], which would indeed not impose these sequences under selection. Our data therefore suggests that in the type VI-B CRISPR locus of *F. columnare,* novel spacers might be acquired by recruiting the acquisition machinery from the adjacent C1 locus, although more specific studies are needed to substantiate this.

Sampling. As random field samples, the bacterial isolates in our study may not represent the complete CRISPR spacer pool present in the sampled fish farm's *F. columnare* population. However, the genotype specific infectivity of the phages allows us to dramatically narrow down the possible phage-bacterium interactions, increasing the possibility that even a smaller sample size, compared to, for example, spacer profiling by deep sequencing, is representative of the spacer pool of the specific *F. columnare* genotype at a given time.

Supplementary References

1. Suomalainen L-R, Kunttu H, Valtonen T, Hirvelä-Koski V, Tiirola M. Molecular diversity and growth features of *Flavobacterium columnare* strains isolated in Finland. *Dieases of Aquatic Organisms* **70**, 55-61 (2006)

2. Leenay RT, Beisel CL. Deciphering, communicating, and engineering the CRISPR PAM. *Journal of Molecular Biology* **429**, 177–191 (2016)

3. Laanto E, Bamford JKH, Ravantti J, Sundberg L-R. The use of phage FCL-2 as an alternative to chemotherapy against columnaris disease in aquaculture. *Frontiers in Microbiology* **6**, 829 (2015)

4. Crooks GE, Hon G, Chandonia JM, Brenner SE. WebLogo: A sequence logo generator, *Genome Research*, **14**,1188-1190 (2004)

5. Lopatina, A. *et al.* Metagenomic analysis of bacterial communities of Antarctic surface snow. *Frontiers in Microbiology* **7**, 398 (2016).

6. Smargon, A. A., *et al.* Cas13b is a Type VI-B CRISPR-associated RNA-Guided RNase differentially regulated by accessory proteins Csx27 and Csx28. *Molecular Cell.* **65**, 618-630 (2017)

7. Swarts, D. C., Mosterd, C., van Passel, M. W. J., & Brouns, S. J. J. CRISPR interference directs strand specific spacer acquisition. *PloS One* **7**, ne35888 (2012)

8. Wang, J., Li, J., Zhao, H., Sheng, G., Wang, M., Yin, M., & Wang, Y. Structural and mechanistic basis of PAM-dependent spacer acquisition in CRISPR-Cas systems. *Cell*, ***163***, 840–853 (2015)
9. Abudayyeh, O. O. *et al.* C2c2 is a single-component programmable RNA-guided RNA-targeting CRISPR effector. *Science* **353,** aaf5573 (2016).
